# Editorial comment to “Relationship between the atrial‐activation pattern around the triangle of Koch and successful ablation sites in slow‐fast atrioventricular nodal reentrant tachycardia”

**DOI:** 10.1002/joa3.13021

**Published:** 2024-03-03

**Authors:** Shiro Nakahara

**Affiliations:** ^1^ Department of Cardiology Dokkyo Medical University Saitama Medical Center Koshigaya Saitama Japan

**Keywords:** catheter ablation, electro‐anatomical mapping, slow pathway modification

Editorial comment on “Relationship between the atrial‐activation pattern around the triangle of Koch and successful ablation sites in slow‐fast atrioventricular nodal reentrant tachycardia.”^1^

Slow‐fast atrioventricular nodal reentrant tachycardia (AVNRT) is the most common type of AVNRT. Catheter ablation of the slow pathway (SP) guided by anatomical information and the local electrograms has long been the gold standard. In the anatomical approach, radiofrequency (RF) energy is applied to the coronary sinus (CS) ostium in the postero‐septal region. In contrast, the electrogram‐guided approach targets sites where SP potentials, such as the Jackman or Haïssaguerre potentials, are recorded in the postero‐septal region anterior to the CS ostium. Despite both well‐established clinical techniques for treatment, the detailed mechanism of the reentrant circuit in AVNRT remains to be elucidated.

Recently, novel methods have been reported for visualizing the estimated SP regions within the triangle of Koch (ToK) based on 3D mapping displays. Bailin et al. reported the usefulness of voltage gradient mapping to estimate the SP region as a bridging area from high‐voltage regions anterior to the tendon of Todaro to low‐voltage regions on the tricuspid annulus.^2^ In further work, methods for estimating the SP regions based on rotating activation patterns within the ToK region accompanied by functional block lines using the latest ultra‐high‐density mapping system have received considerable attention.^3^ However, in a previous study, the activation map covered less than 50% of the tachycardia cycle length of the electrical activity in the ToK during slow‐fast AVRNT. This finding suggests that only a tiny portion of the AVRNT circuit was involved in the ToK.^4^ Thus, this indicates that even current 3D mapping systems have difficulty identifying SP regions during sinus rhythm.

In this issue of the Journal, Watanabe et al. present a novel method to visualize optimal ablation regions using the specific features of the latest mapping system with a small basket catheter with a narrow electrode spacing.^1^ They mapped the region of the ToK during sinus rhythm in 30 patients with AVNRT. In 26 patients (87%), two delayed atrial activation wavefronts collided within the ToK. In addition, spiky potentials preceded the His bundle electrogram in 23 cases (85%) at the wave collision site (whether this potential is a lower nodal bundle [LNB] is still controversial). They also used the LUMIPOINT algorithm to search for optimal treatment sites for AVNRT. The algorithm highlights only spatially and temporally consistent intracardiac electrograms. Therefore, small and spikey potentials, which are ideal target potentials for SP elimination, are not highlighted because they are recorded locally and are not spatially consistent. The algorithm usually considers the highlighted region as the region of interest, which is a near‐field potential. However, the authors have reversed this and focused on the nonhighlight regions as regions of interest, which are far‐field potentials. This idea is considered to be very unique and novel.

The atrioventricular junction region, including the ToK, exhibits local differences in the expression of connexin 45, which is involved in the conduction velocity of the AV node and surrounding tissue.^5^ Its heterogeneous distribution differences are thought to cause unidirectional block and rotational excitation patterns in the ToK during sinus rhythm. Of note in this manuscript is the idea that the authors used the latest high‐density mapping capabilities to show not only the collision site as the source of rotational‐like excitation but also the presumed LNB and surrounding tissue leading to the penetrating bundle, which is assumed to extend deep into the septum, as a nonhighlighted far‐field electrogram. Indeed, their finding that the areas of dull and spiked potentials recorded as far‐field electrograms in the nonhighlighted areas are more likely to be safe and reliable optimal treatment sites, regardless of whether RF or cryoenergy was used, is commendable.

Some limitations include the fact that this study was a retrospective analysis and the number of cases was small. It should also be noted that in four patients (13%), despite careful mapping, no collision points could be identified within the ToK. The authors' methodology cannot be applied to all cases because of the variability in the pattern of electrical excitation propagation within the ToK in each case. Further studies are needed to determine whether effects such as the displacement of the ToK region because of aortic stretch are involved. It may also be necessary to verify the functional changes in the collision site caused by early atrial stimulation and to compare them with actual electrical excitation propagation mapping data during the tachycardia. Finally, whether the unhighlighted regions identified by the authors as target sites are indicative of LNB or inferior nodal extension of the compact node requires further investigation.

The ToK region remains an elusive electrophysiological region, and recent high‐density mapping analyses have attracted much attention. The concept of Watanabe et al. to use the features of the latest mapping system in reverse to focus on nonhighlighted regions suggesting conduction regions downstream of the compact AV node, which would pass through deep septal regions, is novel, timely, and deserves due attention.

## FUNDING INFORMATION

None.

## CONFLICT OF INTEREST STATEMENT

Author declare no conflict of interests for this article.
